# TIM-4 is expressed on invariant NKT cells but dispensable for their development and function

**DOI:** 10.18632/oncotarget.12153

**Published:** 2016-09-20

**Authors:** Xilin Zhang, Jun Gu, Li Zhou, Qing-Sheng Mi

**Affiliations:** ^1^ Henry Ford Immunology Program, Henry Ford Health System, Detroit, MI, United States of America; ^2^ Department of Dermatology, Henry Ford Health System, Detroit, MI, United States of America; ^3^ Department of Dermatology, Changhai Hospital, Second Military Medical University, Shanghai, China; ^4^ Department of Internal Medicine, Henry Ford Health System, Detroit, MI, United States of America; ^5^ Department of Immunology and Microbiology, Wayne State University School of Medicine, MI, United States of America

**Keywords:** TIM-4, invariant NKT cells, development, function, polarization, Immunology and Microbiology Section, Immune response, Immunity

## Abstract

T cell immunoglobulin and mucin-4 (TIM-4), mainly expressed on antigen presenting cells, plays a versatile role in immunoregulation. CD1d-restricted invariant natural killer T (iNKT) cells are potent cells involved in the diverse immune responses. It was recently reported that recombinant TIM-4 (rTIM-4) alone enhanced cytokine production in NKT hybridoma, DN32.D3 cells. Hence, we hypothesized that TIM-4 might regulate iNKT cell biology, especially their function of cytokine secretion. For the first time, we identified that TIM-4 was expressed in thymus iNKT cells, and its expression increased upon iNKT cell migration to the secondary lymphoid organs, especially in lymph nodes. Using TIM-4-deficient mice, we found that lack of TIM-4 did not disturb iNKT cell development, maturation, peripheral homeostasis and cytokine secretion. Moreover, TIM-4 deficiency did not alter the polarization of iNKT sublineages, including NKT1, NKT2 and NKT17. Finally, the mixed bone marrow transfer experiments further confirmed normal iNKT cell development and function from TIM-4-deficient bone marrow. In conclusion, our data suggest that TIM-4 is expressed on iNKT cells but dispensable for their development and function.

## INTRODUCTION

Natural killer T (NKT) cells comprise a unique subset of αβT cells that coexpress a semi-invariant T-cell receptor (TCR) and natural killer (NK) cell-related surface markers. They stringently respond to glycolipid antigens, such as α-galactosylceramide (α-GalCer), presented by the major histocompatibility complex (MHC) class I-like molecule CD1d [1]. Two major types of NKT cells have been described in mice: type I invariant NKT (iNKT) cells are the most abundant subset which express Vα14-Jα18 TCR-α rearrangement together with Vβ8.2, Vβ7, or Vβ2 TCR-β chains, whereas type II NKT cells display heterogeneous TCR αβ chain combinations [2]. Within hours of activation, iNKT cells simultaneously produce large amounts of cytokines, including interferon (IFN)-γ, interleukin (IL)-4 and IL-17, which greatly contribute to diverse immune responses, including antimicrobial immunity, tumor rejection, allergy and autoimmune diseases [3–5]. Therefore, it is essential to understand the mechanisms governing iNKT cell development and function.

iNKT cells derive from CD4^+^ CD8^+^ double-positive (DP) thymus precursors and undergo four distinct stages of differentiation involving sequential downregulation of CD24 and upregulation of CD44 and NK1.1 [6–9]. Firstly, the DP thymocytes, expressing Vα14-Jα18 TCR-α chain as well as signaling lymphocytic-activation molecule (SLAM) co-receptor, are positively selected to become stage 0 iNKT cells (CD24^+^ CD44^low^ NK1.1^−^). Next, stage 0 iNKT cells lower their CD24 expression and turn into highly-proliferative stage 1 (CD24^low^ CD44^low^ NK1.1^−^) and, subsequently, stage 2 (CD24^low^ CD44^high^ NK1.1^−^) iNKT cells. Finally, most of stage 2 iNKT cells leave the thymus, complete maturation and become stage 3 iNKT cells (CD24^low^ CD44^high^ NK1.1^+^) in the peripheral organs, while a minority reside and mature inside the thymus [10]. Recent evidence further modified this notion of sequential developmental process in iNKT cells [11, 12]. Resembling T helper (Th) cells, iNKT cells are at least classified into three polarized sublineages, including NKT1, NKT2 and NKT17, based on their expression of transcription factors T-bet, GATA-3, and RORγt with the distinct secretion of IFN-γ, IL-4 and IL-17, respectively. Despite great advances in understanding the biology of iNKT cells, the detailed underlying mechanisms remain elusive.

The T-cell immunoglobulin domain and mucin domain (TIM) family of genes was first cloned in the T cell and airway phenotype regulator (Tapr) locus as a novel allergy susceptibility gene [13]. It consists of eight members (encoding TIM-1 to TIM-4 and putative TIM-5 to TIM-8) in mice, which are located on chromosome 11B1.1, while three members Tim-1, Tim-3 and Tim-4 in humans are located on chromosome 5q33.2 [14]. All the TIM members share an analogous structure of cell-surface Type 1 membrane protein, containing a N-terminal Cysteine-rich immunoglobulin (Ig) variable-like domain, a mucin-like glycosylated domain, a transmembrane domain and an intracellular tail [15]. Previous studies underscored an essential role of TIM family in diverse immune responses, involving viral infection, allergy, autoimmunity, transplant tolerance and tumor immunity [16–18]. The underpinning mechanisms were principally attributed to their regulation of T cell polarization. High-affinity TIM-1-specific agonist antibody promoted Th1 and Th17 responses, but inhibited regulatory T cell (Treg) differentiation; low-affinity TIM-1 engagement enhanced Th2 polarization with compromised T cell proliferation [19]. And, TIM-2 preferentially promoted Th2 response, while TIM-3 specifically inhibited Th1 differentiation [20, 21].

TIM-4, also named as SMUCKLER (spleen, mucin-containing, knockout of lymphotoxin), was originally identified by gene expression profiling in 2004 [22]. Being the sole TIM member absent in conventional T cells, TIM-4 was mainly expressed in “professional” antigen-presenting cells (APC) under physiological status, including macrophages and dendritic cells (DC) [23–25]. Also, unlike other TIM members, TIM-4 lacks tyrosine phosphorylation motifs on the intracellular tail, which forbids mediating direct signaling of its own [26]. Nevertheless, previous studies demonstrated a pleiotropic immunoregulatory role of TIM-4, functioning as either a costimulatory molecule or as a phosphatidylserine (PS) receptor for apoptotic bodies. As a natural ligand for TIM-1, TIM-4 provoked pre-activated T cell expansion; however, TIM-4 suppressed naïve T cell proliferation by binding to an unknown ligand excluding TIM-1 [23, 27]. Furthermore, TIM-4 regulated the polarization of CD4^+^ naïve T cells towards Th2, Th17 and regulatory T (Treg) sublineages [28–30].

Recently, Kim *et al* discovered that unlike conventional T cells, mouse hepatic iNKT cells and DN32.D3 NKT hybridoma cells constitutively expressed TIM-4 and TIM-1 at a substantial level [31]. Intriguingly, recombinant TIM-4 (rTIM-4) alone, but not recombinant TIM-1 (rTIM-1), enhanced the cytokine production of DN32.D3 NKT hybridoma cells. Moreover, silencing of TIM-4 profoundly lowered IFN-γ and IL-4 secretion by TIM-1-engaged DN32.D3 cells *in vitro*. Hence, we hypothesized that TIM-4 might control iNKT cell biology, especially its function. In this study, for the first time, we uncovered the expression pattern of TIM-4 in iNKT cells located in various lymphoid organs. Using global TIM-4 knockout (KO) mice with a mixed bone marrow transfer model, we demonstrated that TIM-4 was not required for iNKT cell homeostasis, development, maturation and cytokine secretion as well as the polarization of their sublineages.

## RESULTS

### TIM-4 is differentially expressed in iNKT cells from different lymphoid organs

**Figure 1:**
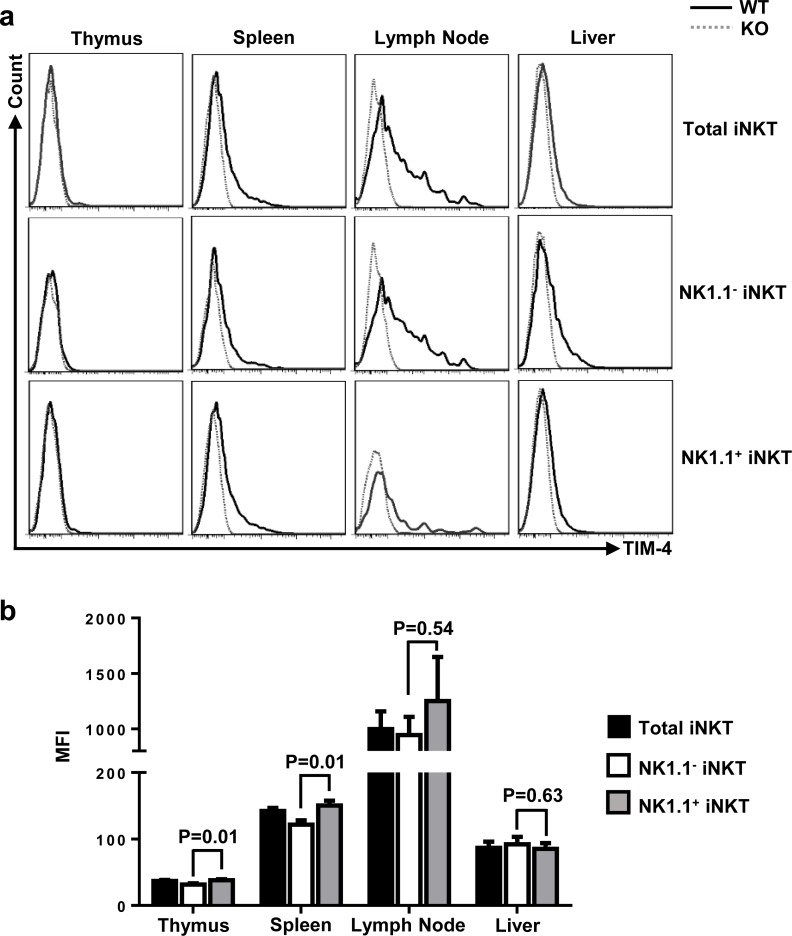
TIM-4 expressions in iNKT cells **a.** The histograms of TIM-4 expressions in the iNKT cells of different lymphoid organs with further evaluation of NK1.1^−^ and NK1.1^+^ iNKT subsets. Solid black histogram represents TIM-4 expression in the iNKT cells from TIM-4 WT mice; and dashed grey histogram represents TIM-4 expression in the iNKT cells from TIM-4 KO mice. Data represent one of three independent experiments, with 4 mice per experiment. **b.** The mean fluorescence intensity (MFI) of TIM-4 expression in the total iNKT cells, NK1.1^−^ and NK1.1^+^ iNKT cell subpopulations of different lymphoid organs from TIM-4 WT mice. Black bar represents total iNKT cells; white bar represents NK1.1^−^ iNKT cells; and grey bar represents NK1.1^+^ iNKT cells. The bar graph (mean ± SEM) represents three independent experiments, with 1-2 mice per experiment.

Consistent with a previous report [24], TIM-4 deficiency did not affect the cellularities of different lymphoid organs (Figure S1). To detect TIM-4 expression in the immature and mature iNKT cells in different lymphoid organs, lymphocytes from thymus, spleen, skin-draining lymph nodes (LN) and liver of TIM-4 KO and wild-type (WT) mice were stained with antibodies against TCR-β, PBS57-CD1d tetramer, NK1.1 and TIM-4. As shown in Figure 1a, thymus iNKT cells barely expressed TIM-4, while the iNKT cells residing at the secondary lymphoid organs exhibited a considerable increase of TIM-4 expression. Among them, LN iNKT cells expressed TIM-4 at the highest level. Compared with their immature counterparts, mature NK1.1^+^ iNKT cells in the thymus, spleen and LNs expressed higher levels of TIM-4, although there was no significant difference between the NK1.1^−^ and NK1.1^+^ subsets of LN iNKT cells (Figure 1b). In a nutshell, TIM-4 is differentially expressed by iNKT cells, based on their location and maturation status.

### TIM-4 is not required for iNKT cell development

**Figure 2:**
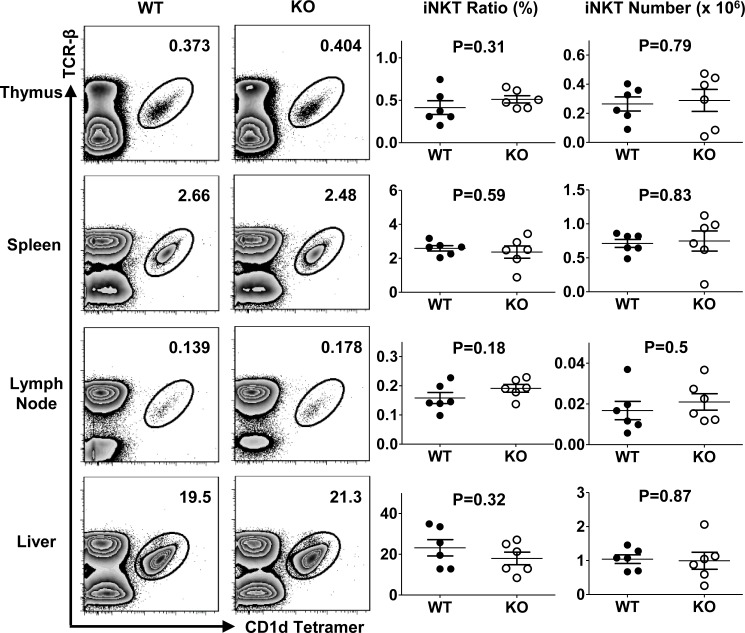
TIM-4 is not required for iNKT cell development Representative dot plots of thymus, spleen, lymph node and liver iNKT cells stained with anti-TCR-β antibody and CD1d tetramer from TIM-4 WT and KO mice (left panel). The frequency (middle panel) and number (right panel) of TCR-β^+^CD1d-tetramer^+^ iNKT cells within different lymphoid organs. Each point represents one individual mouse, and the mean values are indicated by middle horizontal lines from three independent experiments with 4 mice per experiment.

To assess the role of TIM-4 in iNKT cell development, we compared the percentage and absolute number of iNKT cells from TIM-4 WT and KO mice. As shown in Figure 2, lack of TIM-4 did not alter iNKT cell ratio or cell count within any lymphoid organ. Equivalent proliferative and apoptotic rates of iNKT cells from TIM-4 WT and KO mice were also found, which ruled out the possibility that TIM-4 might have an impact on iNKT cell turnover (Figure S2). Therefore, TIM-4 appears to be dispensable for iNKT cell development.

### TIM-4 deficiency does not alter iNKT cell maturation

**Figure 3:**
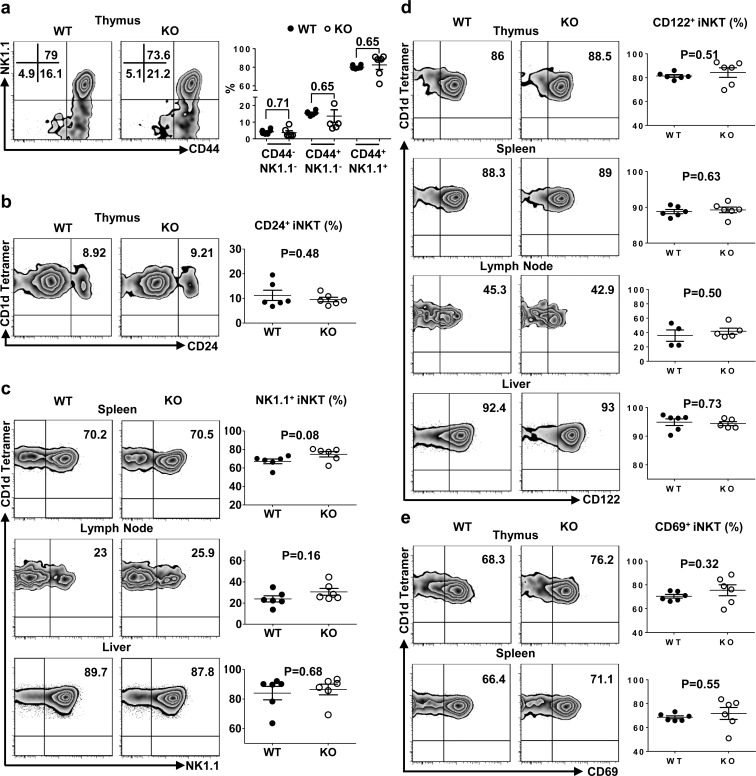
TIM-4 deficiency does not alter iNKT cell maturation **a.** Representative dot plots depict CD44 and NK1.1 expression in thymus iNKT cells of TIM-4 WT and KO mice (left panel). The percentages of iNKT cell subpopulations based on their CD44 and NK1.1 expression patterns gated on thymus TCR-β^+^CD1d-tetramer^+^ iNKT cells (right panel). Black dot represents the percentage of thymus iNKT cell subset from TIM-4 WT mice, and white dot represents the percentage of thymus iNKT cell subset from TIM-4 KO mice. Each point represents one individual mouse, and the mean values are indicated by middle horizontal lines from three independent experiments with 4 mice per experiment. **b.** Flow cytometry analysis (left panel) and percentage (right panel) of CD24-positive thymus iNKT cells from TIM-4 WT and KO mice. Data represent three independent experiments with 4 mice per experiment. **c.** Flow cytometry analysis (left panel) and percentage (right panel) of NK1.1-positive iNKT cells in the spleen, lymph node and liver of TIM-4 WT and KO mice. Data represent three independent experiments with 4 mice per experiment. **d.** Flow cytometry analysis (left panel) and percentage (right panel) of CD122-positive iNKT cells in the thymus, spleen, lymph node and liver of TIM-4 WT and KO mice. Data represent at least two independent experiments with 4-5 mice per experiment. **e.** Flow cytometry analysis (left panel) and percentage (right panel) of CD69-positive iNKT cells in the thymus and spleen of TIM-4 WT and KO mice. Data represent three independent experiments with 4 mice per experiment.

As the expression of TIM-4 in iNKT cells enhanced upon maturation, we explored the possibility that TIM-4 might modulate the maturation process of iNKT cells. As depicted in Figure 3a and 3b, the frequencies of stage 0 to stage 3 iNKT cells within the thymus were all comparable between TIM-4 WT and KO mice. Further analysis of the secondary lymphoid organs showed that the percentage of mature iNKT cells, which expressed NK1.1, remained equivalent between TIM-4 WT and KO mice (Figure 3c). In addition to NK1.1, the maturation of iNKT cells are also accompanied by the upregulated expressions of several other cell surface receptors, such as CD122 and CD69. In accordance with the aforementioned results, TIM-4 deficiency did not alter the frequencies of CD122- and CD69-positive iNKT cells (Figure 3d, 3e). Together, our data suggest that TIM-4 is not required for iNKT cell maturation.

### Normal iNKT cell function in TIM-4 KO mice

**Figure 4:**
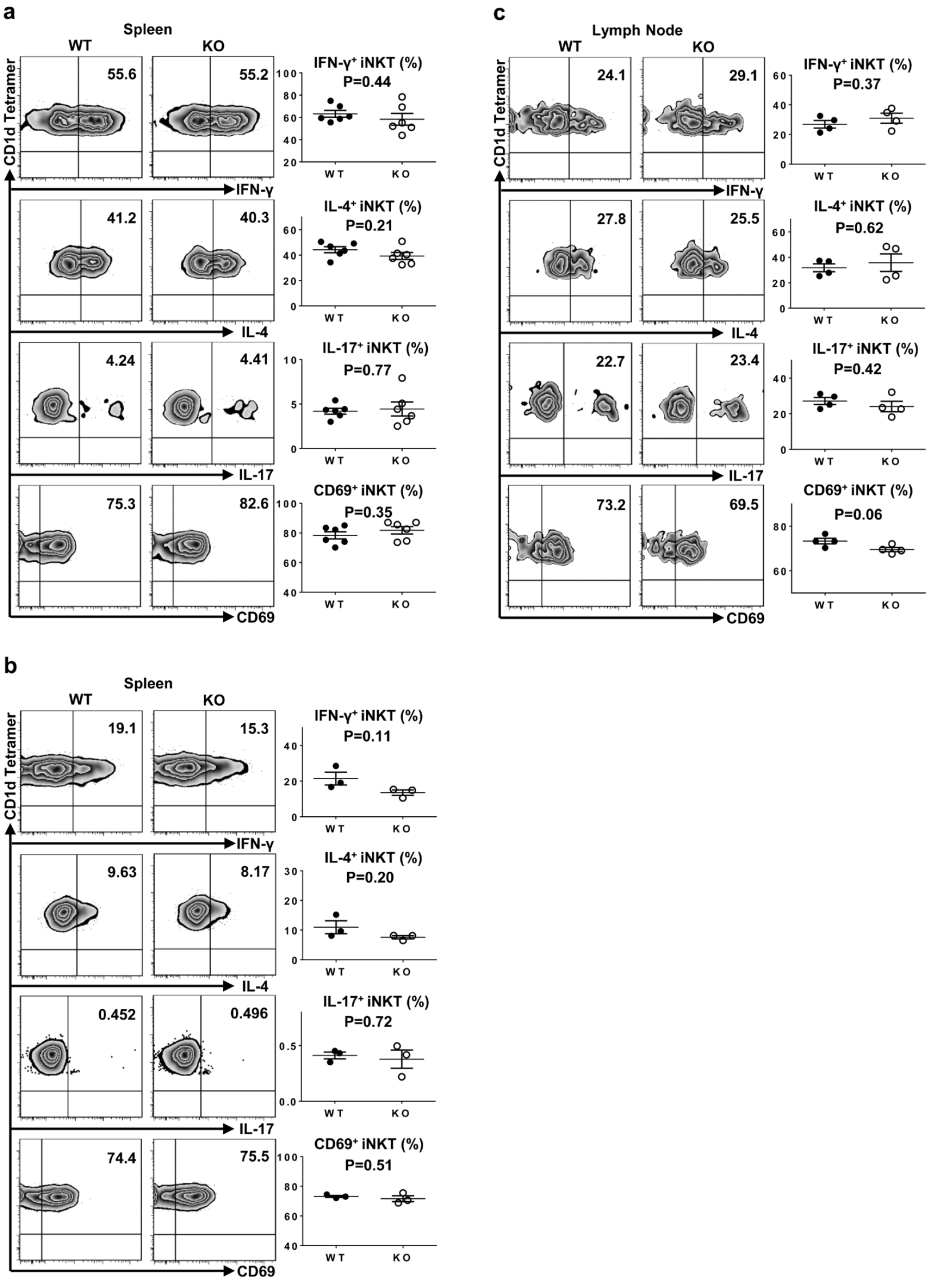
Normal iNKT cell function in TIM-4 KO mice **a.** Representative dot plots of IFN-γ, IL-4, IL-17 and CD69 expressions in spleen iNKT cells of TIM-4 WT and KO mice after PMA and ionomycin treatment *in vitro* for 3 hours (left panel). The percentages of IFN-γ-, IL-4-, IL-17- and CD69-positive iNKT cells (right panel). Each point represents one individual mouse, and the mean values are indicated by middle horizontal lines from three independent experiments with 4 mice per experiment. **b.** Representative dot plots of IFN-γ, IL-4, IL-17 and CD69 expressions in spleen iNKT cells of TIM-4 WT and KO mice after *in vivo* α-Galcer stimulation for 2 hours (left panel). The percentages of IFN-γ-, IL-4-, IL-17- and CD69-positive iNKT cells (right panel). Data represent six mice. **c.** Representative dot plots of IFN-γ, IL-4, IL-17 and CD69 expressions in the lymph node iNKT cells from TIM-4 WT and KO mice after PMA and ionomycin treatment *in vitro* for 3 hours (left panel). The percentages of IFN-γ-, IL-4-, IL-17- and CD69-positive iNKT cells (right panel). Data represent two independent experiments with 4 mice per experiment.

The prompt secretion of a large amount of cytokines upon activation is crucial to the immunoregulatory function of iNKT cells. To explore the role of TIM-4 in iNKT cell function, spleen and skin-draining LN cells were stimulated *in vitro* with phorbol 12-myristate 13-acetate (PMA) and ionomycin for 3 hours, which bypassed proximal TCR-mediated signaling events. The secretion of IFN-γ, IL-4 and IL-17 as well as CD69 expression in both spleen and LN iNKT cells were comparable between TIM-4 WT and KO mice (Figure 4a, 4c). Next, we injected mice with α-GalCer to specifically stimulate iNKT cells *in vivo*. Consistently, 2 hours after α-GalCer treatment, loss of TIM-4 did not affect the cytokine secretions and CD69 expression by spleen iNKT cells (Figure 4b). Hence, TIM-4 deficiency does not alter iNKT cell function.

### Lack of TIM-4 does not affect the polarization of iNKT cell sublineages

**Figure 5:**
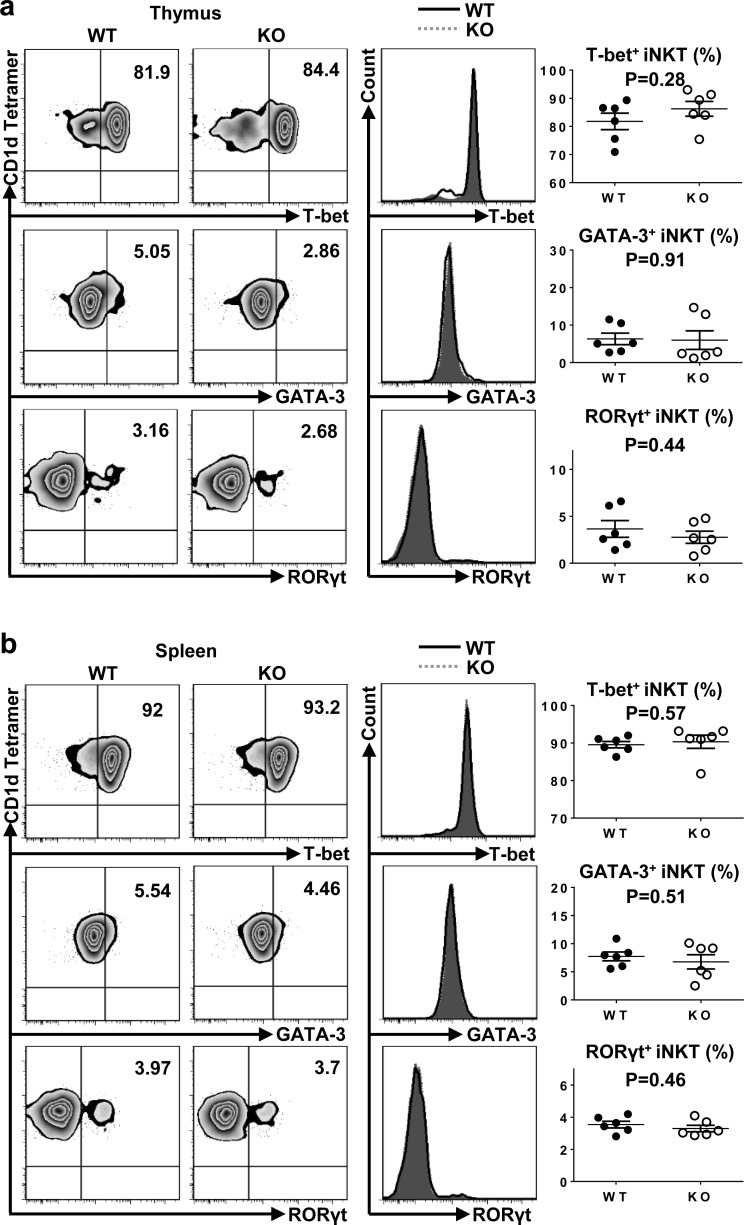
Loss of TIM-4 does not affect the polarization of iNKT cell sublineages **a.** Representative dot plots of T-bet, GATA-3 and RORγt expressions in the thymus iNKT cells from TIM-4 WT and KO mice (left panel). The histograms of T-bet, GATA-3, and RORγt expressions in the thymus iNKT cells (middle panel). Solid black histogram represents the expression of transcription factor in the iNKT cells from TIM-4 WT mice; shaded grey histogram represents the expression of transcription factor in the iNKT cells from TIM-4 KO mice. The percentages of T-bet-, GATA-3- and RORγt-positive thymus iNKT cells (right panel). Each point represents one individual mouse, and the mean values are indicated by middle horizontal lines from three independent experiments with 4 mice per experiment. **b.** Representative dot plots of T-bet, GATA-3 and RORγt expressions in the spleen iNKT cells from TIM-4 WT and KO mice (left panel). The histograms of T-bet, GATA-3, and RORγt expressions in the spleen iNKT cells (middle panel). Solid black histogram represents the expression of transcription factor in the iNKT cells from TIM-4 WT mice; and shaded grey histogram represents the expression of transcription factor in the iNKT cells from TIM-4 KO mice. The percentages of T-bet-, GATA-3-, and RORγt-positive spleen iNKT cells (right panel). Data represent three independent experiments with 4 mice per experiment.

To investigate whether TIM-4 regulated the polarization of iNKT cell sublineages, we examined the expressions of transcription factors in thymus and spleen iNKT cells. As shown in Figure 5a and 5b, both the frequency and mean fluorescence intensity (MFI) of T-bet, GATA-3 and RORγt were equivalent between TIM-4 WT and KO mice. Along with their intact cytokine-secreting function, the proper expression of transcription factors in TIM-4-deficient iNKT cells further confirm that TIM-4 deficiency does not disturb the polarization of iNKT cell subsets.

### Normal iNKT cell development and function in a mixed bone marrow transfer model

**Figure 6:**
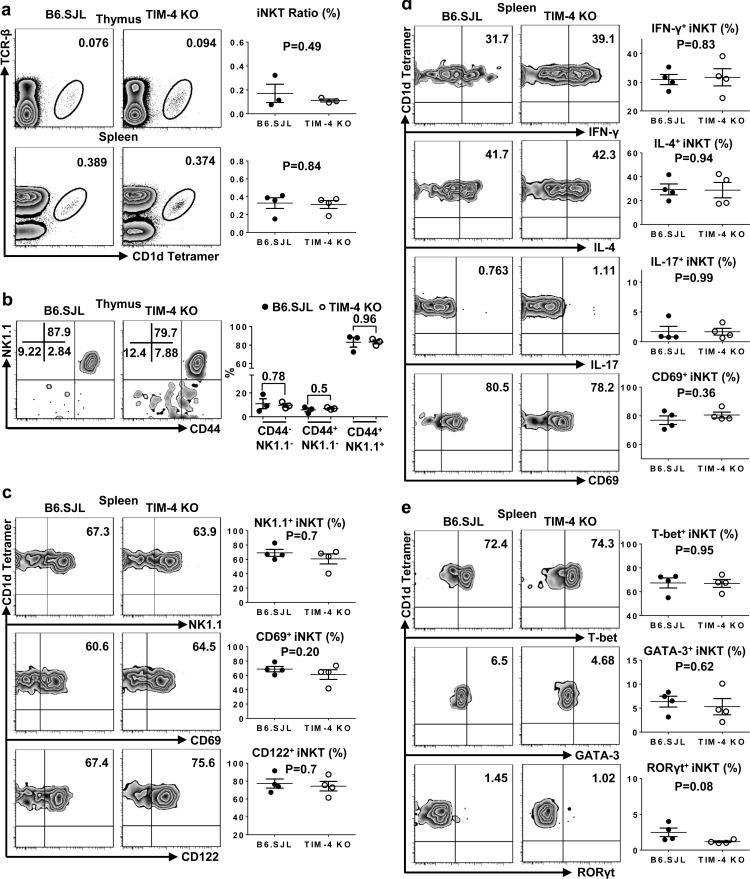
Normal iNKT cell development and function are cell-intrinsic in TIM-4 KO mice The BMs of CD45.2^+^ TIM-4 KO mice and CD45.1^+^ B6.SJL mice were mixed at a 1:1 ratio and transferred into lethally irradiated CD45.1^+^/CD45.2 B6.SJL hosts. Eight weeks later, reconstituted animals were analyzed by flow cytometry. **a.** Flow cytometry analysis (left panel) and frequency (right panel) of thymus and spleen iNKT cells originated from the CD45.1^+^ B6.SJL and CD45.2^+^ TIM-4 KO BMs. **b.** Flow cytometry analysis of iNKT developmental stages gated on the B6.SJL- and TIM-4 KO-derived thymus iNKT cells (left panel). The percentage of each iNKT developmental subset (right panel). **c.** Flow cytometry analysis (left panel) and percentage (right panel) of NK1.1, CD69 and CD122 expressions in the B6.SJL- and TIM-4 KO-derived splenic iNKT cells. **d.** Flow cytometry analysis (left panel) and ratio (right panel) of IFN-γ-, IL-4-, IL-17- and CD69-positive splenic NKT cells from the B6.SJL and TIM-4 KO BMs after PMA and ionomycin treatment *in vitro* for 3 hours. **e.** Flow cytometry analysis (left panel) and frequency (right panel) of T-bet, GATA-3 and RORγt expressions in the B6.SJL- and TIM-4 KO-derived splenic iNKT cells. Data represent 3 to 4 chimeric mice.

The differentiation of iNKT cells and their function are shaped conjointly by both progenitor cells from the bone marrow (BM) and local microenvironment. To further determine whether the normal development and function of TIM-4-deficient iNKT cells are compensated by TIM-4-deficient environmental factors, we utilized a mixed bone marrow transfer model, in which iNKT cell precursors from both TIM-4 WT and KO mice were positioned within the same normal microenvironment. As depicted in Figure 6a, the BM from TIM-4 KO mice reconstituted iNKT cells comparably to that of WT mice. When analyzing NK1.1/CD44 profile in the thymus, equivalent frequencies of developmental iNKT cells were detected between TIM-4 KO and WT BM origins (Figure 6b). Similar results were obtained when assessing the percentages of NK1.1-, CD122- and CD69-positive spleen iNKT cells (Figure 6c). After *in vitro* stimulation with PMA and ionomycin for 3 hours, the production of IFN-γ, IL-4 and IL-17 along with CD69 expression were comparable between TIM-4 WT and KO BM-derived spleen iNKT cells (Figure 6d). Consistently, the expression of T-bet, GATA-3 and RORγt in spleen iNKT cells were also equivalent (Figure 6e). Overall, our BM transferring data further support the notion that TIM-4 is not required for iNKT cell development and function.

## DISCUSSION

TIM-4 was originally identified to be preferentially expressed in peripheral lymphoid tissues, including splenic white pulp and marginal zone, LN subcapsular sinus and paracortex area as well as Peyer's patches [22]. Later on, TIM-4 expression was predominantly detected in typical APCs, including macrophages and dendritic cells, other than conventional T cells [23–25]. To the best of our knowledge, we are the first to report that TIM-4 is differentially expressed in iNKT cells residing at various lymphoid organs, and its expression is generally upregulated in mature iNKT cells.

TIM-4 plays a pleiotropic role in T cell immunity by specific ligations to other molecules expressed on conventional T cells. TIM-4 was initially recognized to be a natural ligand of TIM-1, and *in vivo* administration of TIM-4-Ig fusion protein stimulated TIM-1-expressing pre-activated T cell proliferation [23]. However, Mizui *et al* [27] showed that TIM-4-Ig fusion protein could also bind to other unknown receptors and exert an inhibitory activity on naïve T cells, which did not express TIM-1. In addition, TIM-4 is capable of modulating Th cell differentiation. TIM-4 generally favors a Th2 over Th1 polarization after environmental disturbance [24, 28, 32, 33]. TIM-4-Ig fusion protein suppressed the *in vitro* differentiation of CD4^+^ T cells into the Th17 phenotype [29]. Recently, Yeung *et al* [30] demonstrated that *in vivo* blockade of TIM-4 by antibodies promoted skin allograft survival by conversion of naïve CD4^+^ T cells to allospecific induced Tregs. Altogether, TIM-4 displays a versatile role in regulating T cell immunity even though it is not expressed by conventional T cells.

Previous studies suggest that TIM molecules profoundly impact the biology of iNKT cells. In the presence of TCR stimulation, TIM-1 costimulation on DN32.D3 NKT hybridoma cells enhanced the cellular secretion of IL-4 while preventing the production of IFN-γ [31]. TIM-1 also mediated the binding of hepatitis A virus (HAV) by human liver NKT cells as well as the activation of human NKT cells triggered by HAV [34]. In addition, TIM-1 facilitated pulmonary iNKT cells to bind rather than engulf apoptotic cells as a typical PS receptor, which enhanced iNKT cell production of IFN-γ and IL-4 and subsequent initiation of airway hyperreactivity [35]. On the other hand, ligation of TIM-3 by galectin-9 in hepatic TIM-3^+^ NKT cells led to activation-induced apoptosis and deletion [36]. However, galectin-9 stimulation on Kupffer cells also increased their secretion of IL-15, which would enhance hepatic NKT cell proliferation [36]. Lately, Kadowaki *et al* [37] reported that galectin-9 upregulated the frequency of splenic NKT cells, particularly TIM-3-expressing NK1.1^+^ NKT cells and further IL-17^+^ NK1.1^+^ NKT cells. In contrast with TIM-1 and TIM-3, the role of TIM-4 in NKT cell biology remains largely unexplored.

Recently, Kim *et al* [31] discovered that unlike conventional T cells, both mouse hepatic iNKT cells and DN32.D3 NKT hybridoma cells constitutively expressed TIM-4. Notably, rTIM-4 alone, but not rTIM-1, could enhance the cytokine production of DN32.D3 cells, implying that TIM-4 might play a crucial role in iNKT cell activity [31]. Moreover, silencing of TIM-4 in DN32.D3 cells significantly hampering cytokine secretion induced by TIM-1 engagement along with TCR stimulus, indicating that TIM-4 is also critically involved in the activation of iNKT cells mediated by TIM-1 signaling [31]. Considering that TIM-1 and TIM-4 act as ligands mutually, these results suggested that TIM-4 might serve as an essential costimulatory molecule for iNKT cell activity. In our study, we utilized TIM-4-deficient mice to assess the role of TIM-4 in the biology of iNKT cells, and discovered that lack of TIM-4 does not affect iNKT cell differentiation, maturation or cytokine secretion in a cell-intrinsic manner. The discrepancy between previous data and our results are probably due to: (1) the *in vitro* supplement of rTIM-4 might produce a higher level of TIM-4 exceeding normal physiological range; (2) TIM-4 might be redundant for iNKT cell activity in the absence of TIM-1 engagement; (3) DN32.D3 cells are a Vα14^+^ CD1d-specific NKT hybridoma cell line, which probably differ from normal iNKT cells in some aspects; (4) it is possible that recombinant protein mediated ligation and RNA interference might produce unpredictable off-target effects, which could bring about confounding factors in interpreting the role of TIM-4 in iNKT cell biology. Nonetheless, we cannot rule out the possibility that TIM-4 might participate in the other unexamined properties of iNKT cells, which include their anergy induction [38], infrequent sublineage (NKT10 or follicular helper NKT cells) polarization [39, 40] and disease-associated immune functions [41–43]. Limitations of this study also involve the potential impaired phagocytosis of TIM-4-deficient APCs, which might compromise the CD1d-restricted presentation of α-Galcer to iNKT cells and subsequently disguise their altered cytokine-secreting function [24, 44]. Future studies, especially with the utility of compartment-specific TIM-4 ablated mice, would provide a better insight into these unanswered questions.

In conclusion, we demonstrate that TIM-4 is differentially expressed on iNKT cells according to their location and maturation status. Moreover, lack of TIM-4 does not disturb iNKT cell development, maturation, functions and NKT1/NKT2/NKT17 polarization, indicating that TIM-4 is not a key gene in regulating iNKT cell biology.

## MATERIALS AND METHODS

### Mice

TIM-4 KO mice were described previously [24], and kindly provided by Dr. Vijay K. Kuchroo (Brigham and Women's Hospital, Harvard Medical School).C57BL/6 wild-type mice were purchased from the Jackson Laboratory. Experiments were conducted at 7 to 10 weeks of age, unless otherwise indicated. Mice were housed in a specific pathogen-free barrier unit. Handling of mice and experimental procedures were in accordance with requirements of the Institutional Animal Care and Use Committee.

### Genotyping

TIM-4 KO mice were genotyped using the following PCR primer pairs: TIM-4 FW 5′-TAGCACAGGTTTTGCGTGAC-3′, TIM-4 WT RV 5′-CTCTGGGACCACGAGAGGTA-3′ and TIM-4 KO RV 5′-GCCAGAGGCCACTTGTGTAG-3′. The TIM-4 deletion allele produced a 250-bp PCR product, whereas the wild-type allele resulted in a 400-bp product.

### Flow cytometry and antibodies

Single-cell suspensions were incubated with anti-FcγRII/III (clone 2.4G2) for 10 minutes at 4°C, and subsequently stained for surface and intracellular markers with the conjugated monoclonal antibodies listed as below: Anti-B220 (RA3-6B2), anti-TCR-β (H57-597), anti-CD44 (IM7), anti-NK1.1 (PK136), anti-CD69 (H1.2F3), anti-CD122 (5H4), anti-T-bet (ebio4B10), anti-GATA-3 (TWAJ), anti-RORγt (B2D), anti-CD45.1 (A20), anti-CD45.2 (104), anti-Ki67 (SolA15), anti-IFN-γ (XMG1.2), anti-IL-4 (11B11), anti-IL-17 (ebio17B7) and Annexin V were purchased from BD Biosciences (San Jose, CA, USA) or eBioscience (San Diego, CA, USA). Lipid PBS-57 (an analogue of α-GalCer)-loaded murine CD1d tetramers were provided by the National Institutes of Health Tetramer Facility (Atlanta, GA, USA). Data were analyzed using FlowJo software.

### *In vivo* α-GalCer-induced activation assay

Two micrograms of α-GalCer in 100 μl of PBS was injected into the tail vein. For intracellular cytokine staining, spleen cells were collected 2 hours after injection and cultured in T cell medium (RPMI 1640 with 10% FCS, HEPES, penicillin and streptomycin, pyruvate, nonessential amino acids, L-glutamine and 2-ME). Golgistop was added to a final concentration of 3 mM, and the cells were incubated for an additional hour. Cells were extracellularly stained with anti-TCR-β antibody and CD1d tetramer. IFN-γ and IL-4 expressions were detected by intracellular staining and flow cytometry.

### *In vitro* PMA and ionomycin activation assay

Spleen and LN cells were cultured in T cell medium in the presence of PMA (50 ng/ml) and ionomycin (1 mM) for 1 hour; Golgistop was added to a final concentration of 3 mM; and the cells were incubated for an additional 2 hours. IFN-γ, IL-4 and IL-17 expressions were detected by intracellular staining and flow cytometry.

### Mixed bone marrow transfer experiments

To generate bone marrow (BM) chimeras, 6- to 8-wk-old B6.SJL recipient mice (CD45.1^+^ /CD45.2) were lethally irradiated initially with 950 rad. Donor BMs were harvested from age- and sex-matched B6.SJL (CD45.1^+^) and TIM-4 KO mice (CD45.2^+^) by flushing with a syringe containing sterile basal tissue culture medium. After erythrocyte lysis, mature T cells were depleted by biotin-conjugated anti-mouse CD3 (BD Biosciences) monoclonal antibodies and anti-biotin magnetic beads (Miltenyi Biotec) from BMs from each donor, using an AutoMACS sorter (Miltenyi Biotec). Over 90% of mature T cell depletion was confirmed by flow cytometry. CD45.1^+^ B6.SJL and CD45.2^+^ TIM-4 KO mice BMs were mixed at a 1:1 ratio, and 10 million cells per mouse (in a volume of 100 μl) were then injected into the irradiated recipients *via* tail vein. The chimeras were analyzed 8 weeks after reconstitution.

### Statistical analysis

Statistical analysis was performed with Prism 5.0 (GraphPad Software). The two-tailed Student t test was used. Differences were considered statistically significant when values of *p* < 0.05.

## SUPPLEMENTARY MATERIALS FIGURES


