# Supplementary material for: TIM-4 is expressed on invariant NKT cells but dispensable for their development and function
Source: Oncotarget. 2016 Sep 20;7(44):71099–111. doi: 10.18632/oncotarget.12153 (PMC5340118; doi:10.18632/oncotarget.12153)
Supplement: Supplementary file 1 [file oncotarget-07-71099-s001.pdf]

TIM-4 is expressed on invariant NKT cells but dispensable for their development and function

Supplementary Material

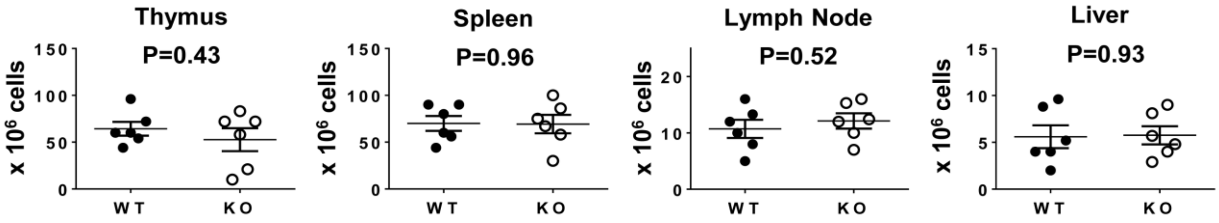

Figure S1: Normal cell numbers of thymus, spleen, lymph nodes and liver in TIM-4 KO mice compared with WT mice. The cell numbers of different lymphoid organs in TIM-4 WT and KO mice. Each point represents one individual mouse, and the mean values are indicated by middle horizontal lines from three independent experiments with 4 mice per experiment.

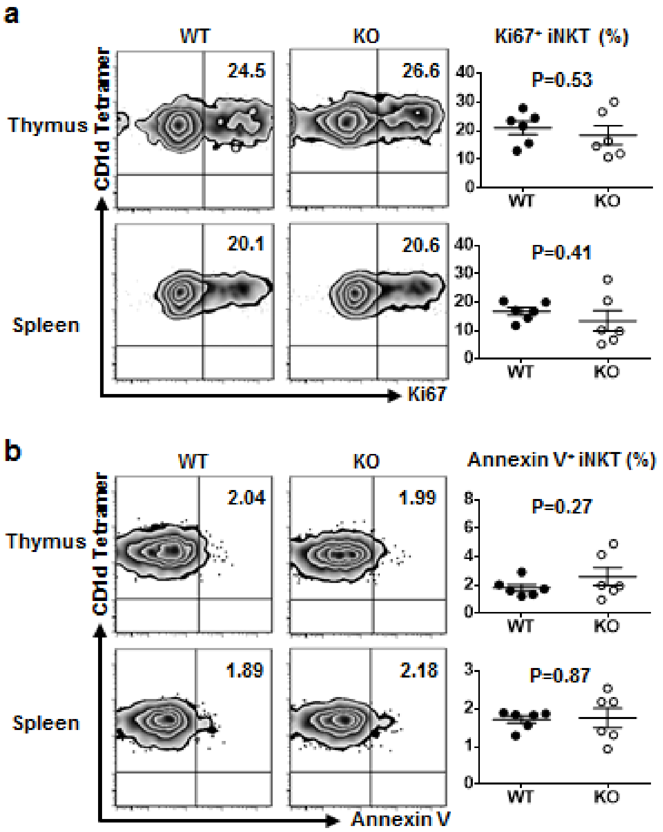

Figure S2: TIM-4 deficiency does not affect thymus and spleen iNKT cell survival. a. Flow cytometry analysis (left panel) and percentage (right panel) of Ki67-positive thymus and spleen iNKT cells from TIM-4 WT and KO mice. Data represent three independent experiments with 4 mice per experiment. b. Flow cytometry analysis (left panel) and percentage (right panel) of Annexin V-positive thymus and spleen iNKT cells from TIM-4 WT and KO mice. Data represent three independent experiments with 4 mice per experiment.
